# Marine Bacterium *Kordia algicida* Reshapes Plankton Microbiome and Induces Metabolomic Rewiring, Independent of Heatwave or Worst-Case Climate Scenarios

**DOI:** 10.1021/acs.jnatprod.5c01435

**Published:** 2026-01-26

**Authors:** Marine Vallet, Mona Staudinger, Kristy S. Syhapanha, Cedric L. Meunier, Inga V. Kirstein, Georg Pohnert

**Affiliations:** † Institute for Inorganic and Analytical Chemistry, Friedrich Schiller University Jena, 07743 Jena, Germany; ‡ Max Planck Fellow Group Plankton Community Interaction, Max Planck Institute for Chemical Ecology, 07745 Jena, Germany; § Department of Chemistry and Biochemistry, University of North Carolina Wilmington, Wilmington, North Carolina 28403, United States; ∥ Center for Marine Science, University of North Carolina Wilmington, Wilmington, North Carolina 28409, United States; ⊥ Alfred-Wegener-Institut, Helmholtz-Zentrum für Polar- und Meeresforschung, Biologische Anstalt Helgoland, 27483 Helgoland, Germany

## Abstract

Marine bacteria are integral components of planktonic communities, where they regulate algal growth, induce cell death, and contribute to bloom termination and species succession. They also play a key role in marine biogeochemical cycling by recycling algal-derived organic matter and releasing bioactive metabolites. Despite their ecological importance, bacterial–plankton interactions and their consequences for community structure and chemistry remain poorly understood. We investigated the impact of the algicidal marine bacterium *Kordia algicida* OT-1 on a natural plankton microbiome collected from a mesocosm experiment simulating present and future climate conditions. Plankton communities were exposed to ambient conditions or to a worst-case climate scenario, with a subset further subjected to a one-week heatwave. After 24 h of incubation, *K. algicida* significantly altered phytoplankton abundance and phylum-level community composition, independent of the applied abiotic conditions. Chemical changes induced by bacterial interactions were assessed by extracting filtrates from cocultures and analyzing them using ultra-high-performance liquid chromatography–high-resolution mass spectrometry (UHPLC-HRMS). Four natural products, i.e., adenosylhomocysteine, two indole alkaloid derivatives, and 5-bromotryptophan, were identified among metabolites released in response to bacterial exposure. Overall, shifts in the planktonic chemical landscape were primarily driven by bacterial activity, rather than abiotic conditions.

## Graphical abstract

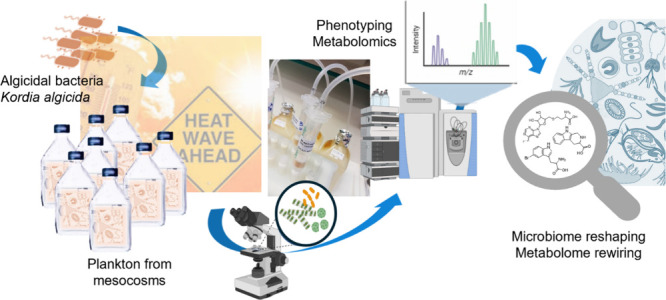

Bacteria are ubiquitous in marine and freshwater ecosystems and engage in complex, multipartite interactions with higher organisms, including algae.^1–3^ This prevalence is primarily due to their remarkable adaptability and rapid evolution, which enable them to produce bioactive metabolites and fulfill diverse ecological roles.^4^ Marine bacteria and their impacts on plankton communities remain poorly understood, despite their abundance and environmental significance in the ocean. Molecular studies have highlighted that these prokaryotes are key drivers in marine ecosystems, playing a crucial role in supporting and regulating the marine food web.^5^ Through their trophic interactions with algae, marine bacteria can promote the growth and development of their hosts.^6^ However, marine bacteria can also adopt a pathogenic lifestyle, killing and lysing algae to release substrates for their own growth.^7^ This transition can result in the termination of a phytoplankton bloom, a crucial ecological process. For example, *Kordia algicida* OT-1, a marine bacterium known for its algicidal properties, produces proteases that induce cell lysis in specific microalgal species.^8^ In laboratory-controlled experiments, the cell-free filtrates of *K. algicida* inhibited the growth of the susceptible diatom *Skeletonema marinoi*, suggesting a chemical mediation.^9^ In mesocosm studies, when introduced to plankton populations recovered from diatom blooms in the North Sea, *K. algicida* caused the rapid decline of *Chaetoceros socialis*. At the same time, competing resistant algae, such as *Phaeocystis*, benefited from this interaction by colonizing newly available ecological niches.^10^ Another example is the marine bacterium *Phaeobacter gallaeciensis*, a member of the Roseobacter group that can switch to a pathogenic mode and secrete potent algicides, known as roseobacticides, in a process that is induced by *p*-coumaric acid, a breakdown product of lignin from aging algae.^11^ Therefore, these bacteria can influence community composition, ultimately contributing to species turnover during phytoplankton bloom seasons; however, little is known about the impact of environmental conditions and heatwaves on bacterial-algal interactions. In the context of possible future climate change scenarios, the Representative Concentration Pathway (RCP) 8.5 scenario represents a high greenhouse gas concentration trajectory that projects significant global warming, accompanied by corresponding shifts in ocean temperature, pH, and nutrient dynamics.^12^ These global changes may also include more frequent and intense heatwaves, which could affect plankton and marine bacteria, potentially altering the frequency, intensity, and composition of algal blooms.^13,14^ Warming temperatures and altered ocean chemistry could impact the physiological responses of bacteria and algae, leading to shifts in the species composition. Changing conditions can potentially enhance the pathogenic potential of specific bacterial strains, such as the model organism *K. algicida*.^15^ Given the importance of these interactions in regulating plankton community structure, understanding how marine bacteria and the plankton microbiome respond to such climate stressors is crucial for predicting future shifts in marine ecosystem dynamics and their associated biochemical processes. In this study, we investigated the effects of introducing the well-characterized marine bacterium *K. algicida*, with known algicidal properties, into natural populations that have experienced different heat waves or climate scenarios. Therefore, the plankton microbiome for these incubations was sampled from a mesocosm experiment that assessed the potential impact of marine heatwaves on plankton communities under ambient or future environmental conditions predicted by the worst-case climate scenario.^16,17^ In addition to monitoring the changes in community composition, we employed metabolomics to document the accompanying metabolic shifts.

## Results and Discussion

### Effect of Algicidal Bacteria on Cell Abundance, Chlorophyll Fluorescence, and Phylum Composition

For this study, plankton microbiomes from the North Sea were enclosed in mesocosms, which were maintained at ambient conditions or exposed to a one-week heatwave followed by one-week of returning to environmental conditions. A subset of the samples was also treated at +3 °C, with a pH reduction of −0.3 pH units, and with 1000 ppm pCO_2_ supplementation to simulate the effects of a possible future climate scenario.^16^ Details about this September 2021 campaign can be found in Ahme et al.^17^ From these mesocosms, plankton samples were collected at the end of the experiment to study the impact of algicidal bacteria on the community composition and metabolism. This resulted in eight conditions, including samples with *K. algicida* added to ambient, heatwave, or RCP 8.5 mesocosm samples, and control mesocosm samples without bacterial addition (Figure 1). The addition of *K. algicida* to the plankton microbiome significantly decreased the overall cell abundance (Figure 2a). These significant changes in total cell abundance (RCP 8.5 scenario (Student’s test, *p* < 0.001) and the ambient conditions (Student’s Test, *p* < 0.05)) were of similar magnitude for all bacteria-treated mesocosm samples, regardless of the heatwave or the conditions of the worst-case RCP 8.5 scenario (Student’s Test, *p* = ns, Figure 2a). A significant decrease in chlorophyll *a* fluorescence was observed in all bacteria-treated mesocosm samples across all environmental conditions tested (Figure 2b, *p*-value < 0.001 for samples in RCP and heatwave scenario, *p*-value < 0.005 for samples in the ambient condition). Heatwave also triggered an increase in chlorophyll *a* fluorescence for bacteria-treated mesocosm samples (Figure 2b, *p*-value < 0.05). Upon further examination of the phytoplankton microbiome composition, bacterial treatment led to the disappearance of several clades, including Cryptophyta, Chlorophyta, Ochrophyta, and Dinophyta (Figure 2c). Only the Bacillariophyta clade remained after 24 h of cocultivation with *K. algicida*, primarily comprising diatom species, as observed mainly for two of the four biological replicates. When we examined heterotrophic flagellated planktonic microbes, including the Euglenozoa, Ciliophora, and Telonemia clades, we followed their disappearance in samples treated with bacteria (Figure 2d), suggesting that the addition of *K. algicida* may induce cell lysis or may stimulate native coexisting bacteria that can lyse plankton. *K. algicida* is notorious for such massive changes: recently, genomic studies identified a single bacterial strain of this species that bloomed during a population-wide crash of the diatom *Phaeodactylum tricornutum* grown in outdoor ponds.^18^ The sequencing analysis supported the finding that 93% of the bacterial community during the demise of *P. tricornutum* belonged to the genus *Kordia*. Furthermore, in a natural algal community, *K. algicida* can influence species abundance and taxonomic composition.^10^ In laboratory-controlled experiments, a broad activity spectrum has been reported for this bacterium, with some diatom species, such as *Skeletonema marinoi*, being susceptible, and others, like *C. didymus*, being resistant.^19^ Therefore, heterotrophic bacteria, such as *K. algicida*, can shape microbial communities in various habitats, including outdoor algal biofuel ponds and natural coastal areas. Here, we also extend the activity spectrum of *K. algicida* to other taxonomic clades, including nonalgal heterotrophic flagellated planktonic microbes, such as Euglenozoa, Ciliophora, and Telonemia. These often-underappreciated clades, which harbor many hard-to-cultivate microorganisms, may be highly susceptible to the lytic activity of *K. algicida*. Further studies should investigate whether the released protease is active against these microbial clades and if these effects are chemically mediated through the addition of cell-free filtrates.

**Figure 1:**
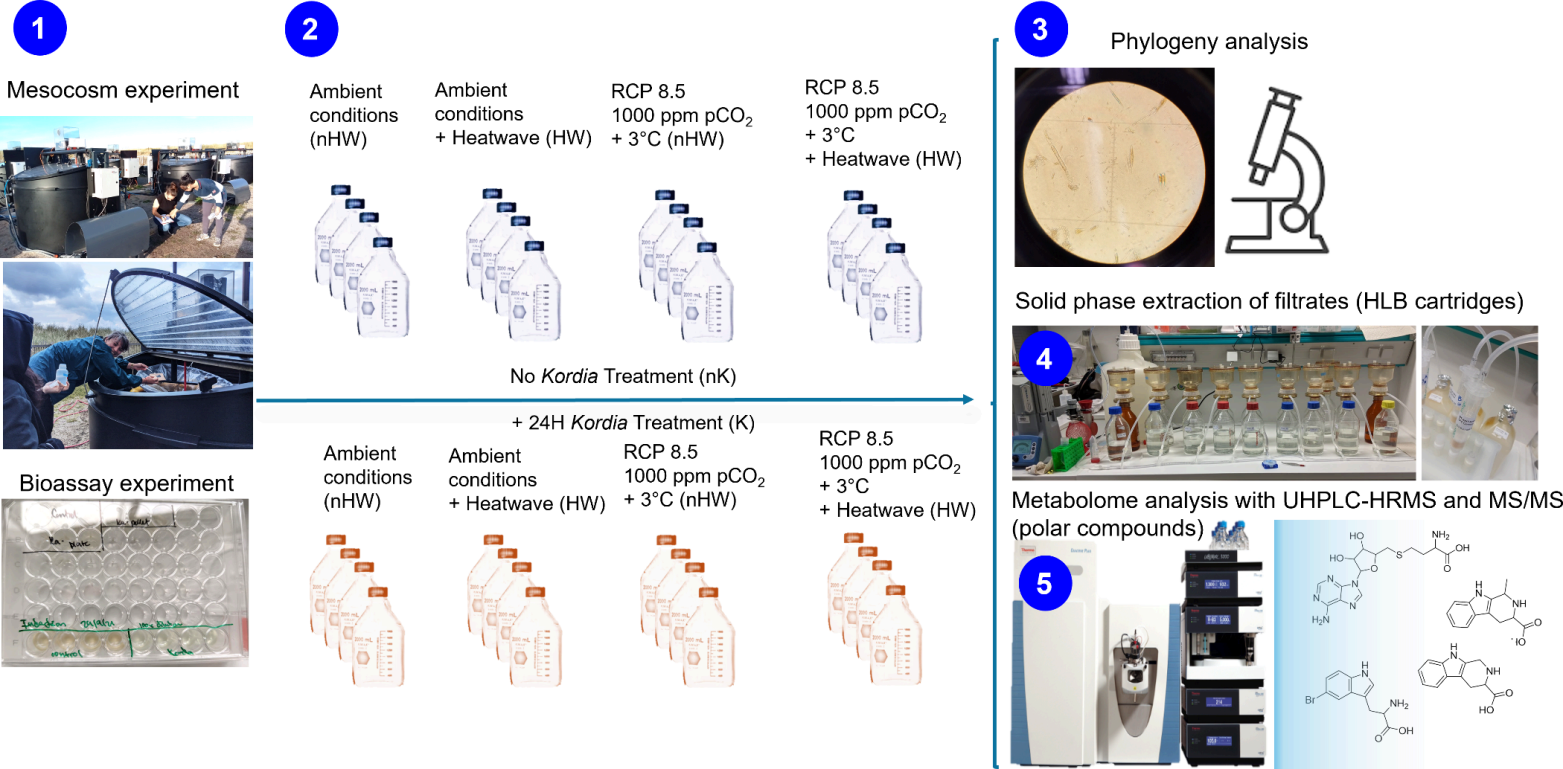
Experimental design and workflow of the study of bacteria treatment on plankton mesocosm samples subjected to ambient conditions, environmental stress, and heatwave. (1) A 1-month length mesocosm experiment was conducted, including a 1-week heatwave treatment, in outdoor, semi-enclosed tidal tanks that mimic coastal conditions. In parallel, the algicidal effect of *K. algicida* was confirmed on the diatom *S. marinoi* in bioassays. (2) Mesocosm samples were retrieved, and some were treated for 24 h with *K. algicida*. (3) Phylogeny analysis based on morphological criteria was done on all mesocosm samples to determine the phylum composition. (4) Solid phase extraction of the filtrates from all mesocosm samples was conducted using HLB cartridges to recover extracts. (5) The extracts were analyzed with UHPLC-HRMS to obtain exometabolome profiles of bacterium-treated and untreated mesocosm samples. The polar metabolites were analyzed by tandem mass spectrometry to identify the dysregulated compounds.

**Figure 2:**
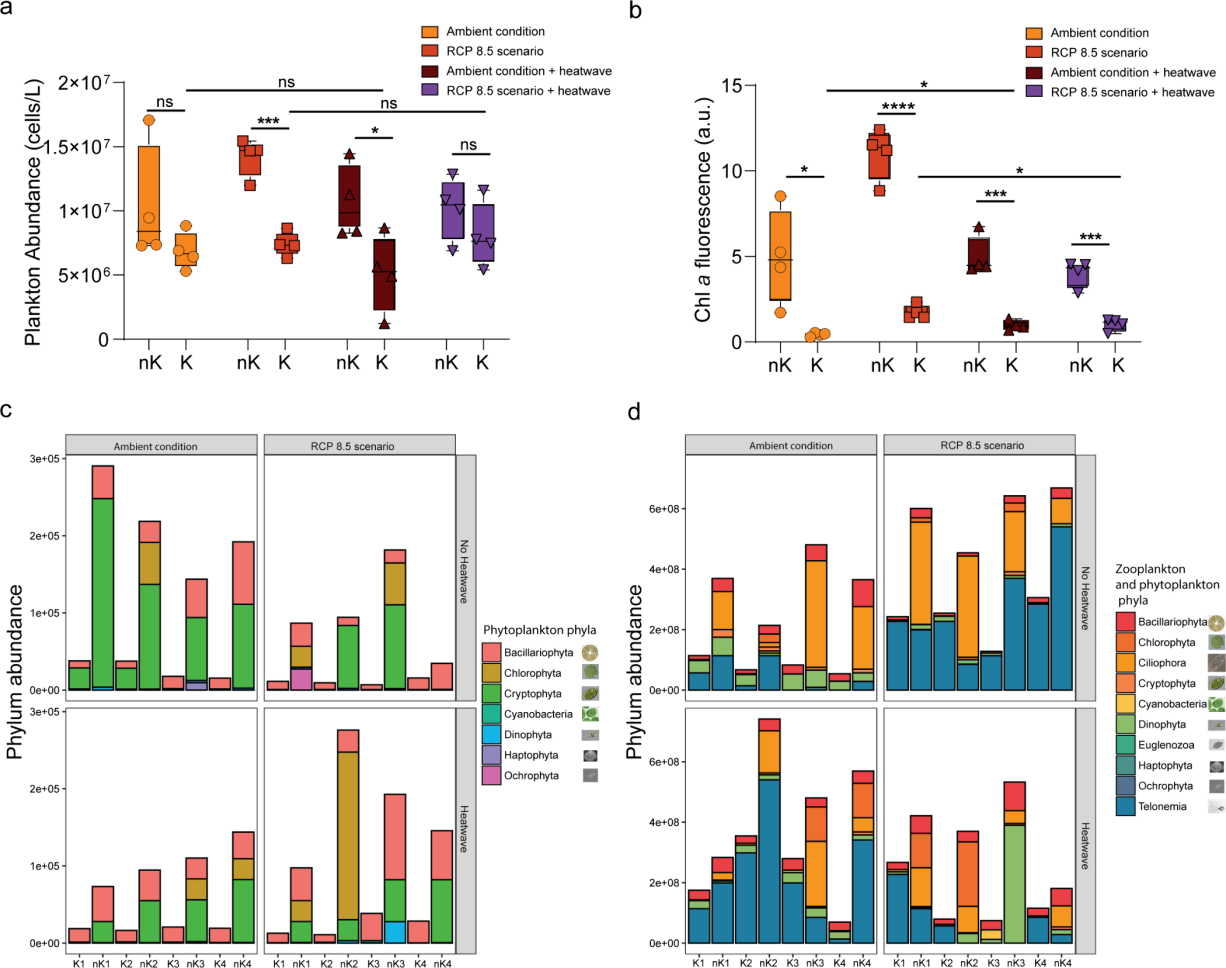
Bacterium *Kordia algicida* alters the cell abundance and phylum composition of the plankton community after 24 h of cocultivation. (a) Significant reduction in cell abundance was observed for mesocosm samples treated with *K. algicida* (K), also when subjected to conditions of the worst-case climate scenario or to ambient conditions with a prolonged 1-week heatwave. Plankton microbiomes untreated with the bacterium are labeled by (nK). (b) Significant decrease in chlorophyll *a* fluorescence following 24 h-incubation bacterial treatment (K) compared to untreated samples (nK) was measured for plankton incubated in all environmental conditions. Statistical significance was tested using unpaired Student’s Test (*P*-value ****p < 0.0001, ***p < 0.001, *p < 0.05, ns nonsignificant). (c) 24-h treatment with the bacterium *K. algicida* (K) modified the phylum abundance (cells per mL) and composition of the phytoplankton community for all environmental conditions tested (Numbers 1, 2, 3, 4 refer to individual measurements/biological replicates). (d) A similar alteration was revealed for phyla, including heterotrophic and nonphototrophic flagellated organisms, including Ciliophora, Euglenozoa, and Telonemia.

### *Kordia algicida* Induces the Release of Metabolites in the Plankton Microbiome

We investigated whether *K. algicida* influences the plankton metabolome and whether this process involves chemical signaling via the release of low-molecular-weight metabolites. These questions were assessed by performing solid-phase extraction and exometabolome profiling of plankton supernatants using ultra-high-performance liquid chromatography coupled with high-resolution mass spectrometry (UHPLC-HRMS). Using HLB cartridges, we aimed to recover and analyze the polar to medium-polar compounds often associated with algal responses to abiotic stresses, such as choline, ectoine, and proline.^20,21^ After removing features from the seawater blank samples, the exometabolome analysis yielded a data matrix of 1,098 features for the positive polarity and 545 features for the negative polarity, annotated by putative elemental composition, *m*/*z* adduct, and retention time. We focused on the data set with negative polarity, which yielded more spectral matches with substances in public databases. Principal Component Analysis (PCA) of the data revealed that the metabolic profiles of the untreated plankton microbiome were distinct from those of the bacteria-treated plankton, regardless of the environmental conditions (heatwave or worst-case climate scenario). In this analysis, the Heatwave treatment did not significantly alter the exometabolome of the plankton microbiome or the response to *K. algicida*. Metabolic profiles of released filtrates from the plankton microbiome under ambient conditions and under the worst-case climate scenario were substantially discriminated in the PCA, with total explained variances of 47.3 and 49.2%, respectively (Figure 3a,b). The 24 h coincubation with *K. algicida* bacteria drove separation (PC1), leading to the release of several metabolites from the exometabolome of mesocosm samples under worst-case climate conditions (Figure 3c). These could originate from either the plankton microbiome or the bacterium *K. algicida*. A discriminant analysis identified 54 significant features with significantly altered abundance between bacteria-treated mesocosm samples and untreated samples (Figure 3b). By further examination of these significant features, four metabolites were detected only in bacteria-treated exometabolomes, regardless of the environmental conditions (Figure 3c). Using MS/MS experiments in both polarities, we assigned identities to the four metabolites with a high confidence level 1 (according to Schymanski et al.^22^) by matching their fragmentation patterns to those of purchased analytical standards (Figure 3e). S-Adenosylhomocysteine (SAH), two indole alkaloid derivatives, and 5-bromotryptophan were thus fully identified based on their diagnostic fragments (Figure 3e, Supplementary Data File 1). Moreover, data mining of the most significant metabolites overabundant in bacteria-treated samples revealed other significantly altered compounds in both positive and negative modes, of which 44 MS2 spectra were available and were further analyzed using SIRIUS and GNPS (Supporting Information File 1). Among these significant metabolites specific to bacteria-treated samples were putatively identified tryptophan derivatives, including cyclomethyltryptophan (Supporting Information, Data File 1). Another alkaloid, putatively related to deoxycytochalasin H, was identified in the positive mode. Additionally, several oligopeptides, peptides, fatty acids, alpha-amino acids, and derivatives were also putatively identified (Supporting Information, File 1).

**Figure 3:**
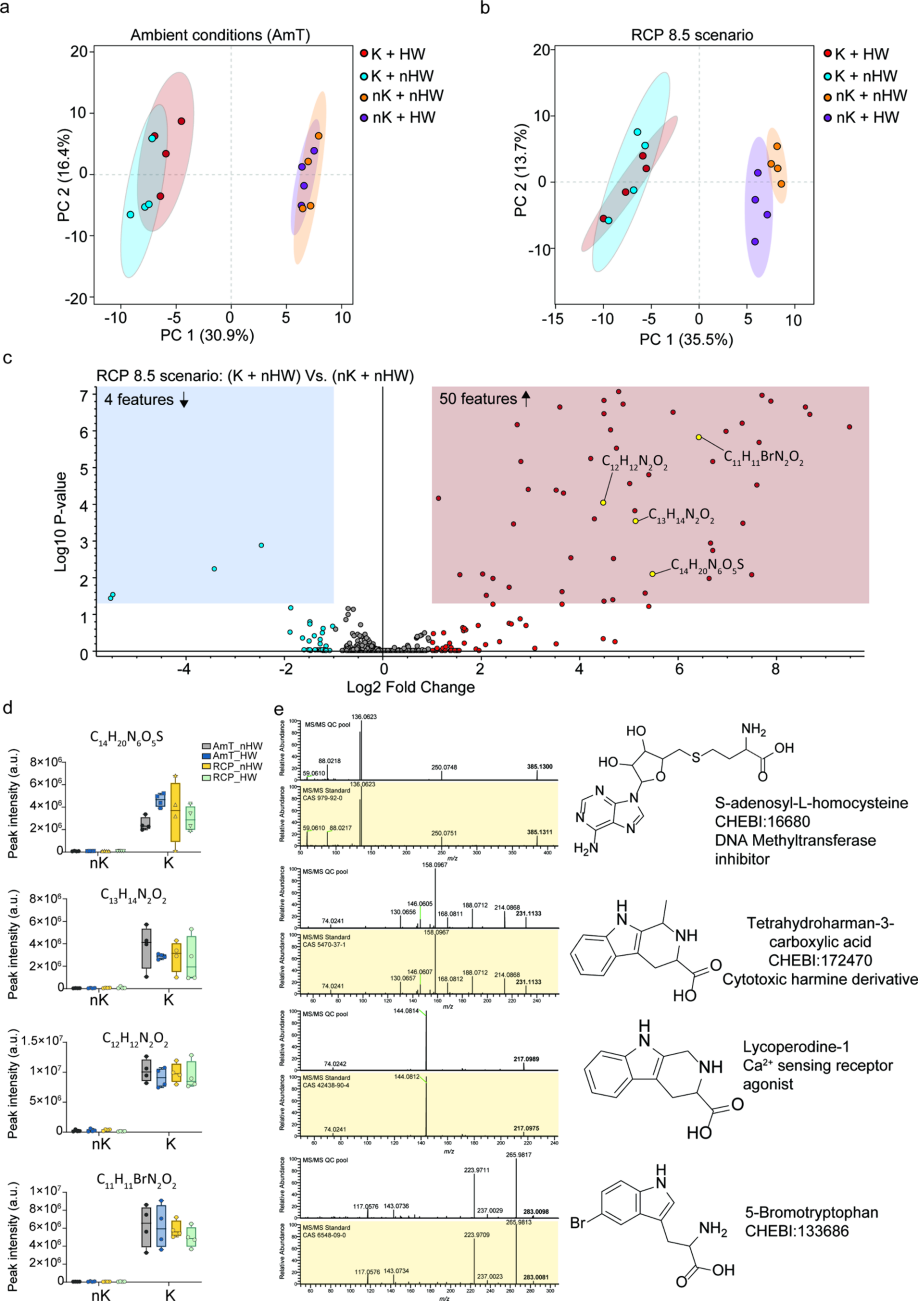
Exometabolome of the plankton microbiome is substantially altered by 24-h treatment with *K. algicida* (K), compared to the untreated plankton microbiome (nK), regardless of the mesocosm conditions (a) ambient or (b) under the worst-case climate scenario. (c) Volcano plot showing the 54 features found by Student’s test that found significantly altered abundance between plankton treated with *K. algicida* (K) Vs Untreated plankton (nK) for samples subjected to the worst-case climate scenario and untreated + heatwave (nHW). Among these, four features (in yellow) were further examined. (d) The four features were all found to be associated explicitly with the exometabolome profiles of bacteria*-*treated plankton (K), and none were detected in the exometabolome profiles of untreated ones (nK). (e) MS/MS fragmentation experiments enabled the identification of the four significant features with unambiguous spectral similarity matches between fragments from the QC pool sample and analytical standards. We identified S-adenosylhomocysteine, two indole alkaloid derivatives, and 5-bromotryptophan as the released substances detected in mesocosm samples treated with *K. algicida* across all conditions tested.

The metabolite *S*-adenosylhomocysteine (SAH) was detected in bacteria-treated exometabolomes and is often reported as a marker of bacterial metabolic activity.^23^ SAH was also detected in extracts from the supernatant of monocultures of *K. algicida* and in cocultures of the bacteria with the coccolithophore *Gephyrocapsa huxleyi* under controlled laboratory conditions.^24^ SAH is the biosynthetic precursor of homocysteine and is involved in bacterial *quorum-sensing* signaling, serving as an intermediate.^25^ Indeed, SAH is a product of *S*-adenosylmethionine (SAM)-dependent methylation reactions and is toxic; in cells, it is converted to homocysteine and adenosine by the enzyme SAH hydrolase.^26^ SAH is involved in the activated methyl cycle, which drives the formation of methionine and its subsequent conversion to SAM. SAH acts as a competitive inhibitor of methyltransferases in eukaryotic cells, while SAM is used to methylate substrates by these enzymes.^27^ This inhibitory effect maintains the balance between SAM and SAH levels, so if SAH builds up, DNA methylation activity decreases. DNA methylation is crucial for bacteria and algae, as it is a key epigenetic mechanism that regulates gene expression, enables defense against pathogens,^28^ and regulates stress responses.^29^ Some metabolites identified by the GNPS analysis were cross-confirmed within the SIRIUS platform, including two indole alkaloids, e.g., 1,2,3,4-tetrahydroharmane-3-carboxylic acid (Supplementary Data File 1). The two identified indole alkaloids act as agonists of Ca^2+^ sensing receptors in eukaryotic cells.^30^ Hence, they are frequently reported for their cytotoxicity against various cell types. Indole alkaloids have been primarily reported as phytochemicals in higher plants; however, they can also occur in marine algae.^31^ Indole alkaloids, including norharman, are produced by diatoms and are induced during parasite cell infection by intracellular obligate oomycetes.^32^ The identification of which plankton microbes produced the indole alkaloids in our experiments and their potential role in marine microbial interactions remains to be determined.

Among the identified low-molecular-weight metabolites in the bacteria-treated plankton microbiome, 5-bromotryptophan is a nonproteinogenic alpha-amino acid that is often found as a structural element in secondary metabolites of marine sponges and lower marine invertebrates, which use it as a component of peptides, indole alkaloids, macrocycles, and other bioactive molecules.^33^ Here, this substance may be a product of direct amino acid bromination or a breakdown of more complex natural products. Future studies on these identified metabolites should determine their origin as a natural product produced by the plankton microbiome, the bacterium *K. algicida*, or other bacteria in the community.

## Conclusion

In summary, we investigated the impact of the marine bacterium *K. algicida* OT-1, a well-established model organism known for its algicidal activity against various algal species, on a natural plankton microbiome that was exposed to ambient conditions, a 1-week heatwave, and the worst-case climate scenario in a mesocosm experiment In all treatments, *K. algicida* induces significant changes in the phylum-level composition of the microbiome after 24 h of incubation. We identified the four known natural products S-adenosylhomocysteine, 5-bromotryptophan, and two indole alkaloid derivatives, which are exclusively found upon treatment with the algicidal bacterium. These compounds were detected in all mesocosm samples treated with bacteria, regardless of the climate scenario.

Our findings underscore the significant influence of marine bacteria on the plankton microbiome, demonstrating that *K. algicida* alters the phylum-level composition of the microbiome and induces metabolic changes in the plankton chemical landscape across the environmental conditions tested. These compounds may have putative biological activity against marine microorganisms, and their potential role in mediating plankton interactions should be investigated.

## Experimental Section

### General Experimental Procedures

Anhydrous methanol (Acros Organics) was used as the solvent for the synthesis. Methanol HiPersolv LC-MS grade (VWR Chemicals) was used as the solvent for extraction. Acetonitrile LC-MS grade (Th. Geyer GmbH) and water LC-MS grade (Th. Geyer, GmbH), ammonium acetate LC-MS grade (Merck), and formic acid LC-MS grade (Thermo Scientific) were used as the mobile phases for LC-MS analysis. Analytical separation and metabolome analysis were achieved by using a Dionex Ultimate 3000 UHPLC system (Thermo Scientific) connected to a Q-Exactive Plus Orbitrap mass spectrometer (Thermo Fisher Scientific). Separation was performed using a SeQuant ZIC-HILIC column (2.1 × 150 mm, 5 μm) coupled with a SeQuant ZIC HILIC guard column (2.1 × 20 mm, 5 μm) (Merck). Electrospray ionization was conducted in positive polarity with the following parameters: capillary temperature, 380 °C; spray voltage, 3000 V; sheath gas flow, 60 arbitrary units; and aux gas flow, 20 arbitrary units.

To assess phytoplankton chlorophyll *a* concentration, spectral fluorometry was used at 685 nm (AlgaeLabAnalyzer, bbe Moldaenke GmbH, Schwentinental, Germany). An inverted microscope (Olympus CKX41; Olympus Scientific Solutions) was used to visualize microorganisms.

### Experimental Biological Setup

The plankton microbiome in this study was sampled during a mesocosm experiment conducted over 3 weeks in the mesocosm facilities at the Wadden Sea station of the Alfred Wegener Institute Helmholtz Centre for Polar and Marine Research on the island of Sylt, Germany, in September 2021.^16,34^ Using a multiple driver approach and based on the predictions by the Intergovernmental Panel on Climate Change for the end of the 21st century (IPCC), temperature and pCO_2_ levels were chosen to represent (1) ambient conditions (condition observed in the field in real time; *T*: 18.4 ± 0.3 °C; pH: 8.3 ± 0.1), and (2) a severe global change heat scenario based on RCP 8.5 scenario (RCP 8.5; +3 °C, −0.3 pH, 1000 ppm pCO_2_). Additionally, a subset of samples was subjected to a 1-week heatwave, initiated 7 days after the initial inoculation of the mesocosm bags. Then, these samples were left for an additional 2 weeks under the initial conditions to test the community’s resilience. We sampled 2 L from the mesocosm bags for this study, 30 days after the initial inoculation. The sample was then filtered through a 100 μm sterile nylon mesh to exclude mesozooplankton and copepod grazers. The 2 L plankton mesocosm samples were split evenly into two 1 L Glass Schott bottles. One bottle was kept as is (untreated, nK), and the other was inoculated with bacteria (bacteria-treated, K). *K. algicida* strain OT-1 was grown in liquid broth medium (Marine broth, 2216, Millipore) for 24 h. Shortly before addition, the cultures were washed twice with filtered seawater by centrifugation (15 min at 12,000 rpm) in 50 mL flasks (Sarstedt). The washed *K. algicida* cultures were diluted with seawater to an optical density (550 nm) of 1, corresponding to about 10^7^ cells per mL. Ten mL of this was added to each of the 1 L plankton cultures to achieve an optical density of 0.01, resulting in a lower bacterial cell density more like natural ecological conditions. Ten mL of filtered seawater was added to the control cultures. The plankton cultures were then incubated for 24 h under either ambient conditions or RCP 8.5 conditions (abbreviated as EIT or RCP in raw files), and the samples were subjected to a 1-week heatwave (abbreviated as HW in raw files). As additional controls, three 1L Schott bottles containing filtered seawater with *K. algicida* and one 1L Schott bottle containing filtered seawater only were each incubated at both temperature groups and later extracted in the same manner to serve as blanks to be subtracted. This experiment with bacteria-treated mesocosm samples was conducted with four independent biological replicates.

The algicidal activity of *K. algicid*a was assessed by the addition of *K. algicida* (0.5 mL of OD_620 nm_ culture) to a culture of susceptible *S. marinoi* in parallel with the bacterial treatment of mesocosm samples. Algicidal activity was confirmed visually by the death of *S. marinoi* cultures after 24 h treatment of 0.1 mL in 2 mL of algal cultures grown in 24-well plates (all cells were lysed, and cellular debris was observed by microscopy).

### Cell Abundance, Phylum Composition, and Statistical Analysis

To determine phytoplankton composition, 50 mL of each mesocosm sample was recovered twice: once before and once after bacterial treatment. These mesocosm samples were mixed with 2 mL of neutral Lugol’s solution in a brown Falcon tube (Sarstedt), stored in the dark, and analyzed according to the method described by Utermöhl.^35^ Planktonic organisms were identified to the species level or grouped by size and shape when species identification was not possible. The analysis was carried out by AquaEcology GmbH & Co. KG in Oldenburg. The algae were sorted by eye (Hugo Moreno), and species/phyla identification was based on morphological criteria seen by microscopy. The full list of Phytoplankton/Protozooplankton identified and sorted by the fee-for-service community structure analysis is available in Supplementary Data file 1. Samples were normalized by the weighted volume.

Data visualization and statistical analysis were performed in GraphPad Prism version 10.0.0 for Windows (GraphPad Software, Boston, Massachusetts, USA, www.graphpad.com). The phylum abundance plots were made using the Python programming language (Python Software Foundation, https://www.python.org/).

### Metabolic Extraction

Samples were processed for metabolic profiles at *T* = 24 h after bacterial treatment. The entire 1 L sample was filtered through GF/C filters (Whatman) and subsequently through 0.2 μm Isopore filters using a filtration unit (VWR International) to remove cells and isolate the released exometabolome. These filtrates were then processed by using solid-phase extraction (SPE) to obtain exometabolomes. HLB-SPE cartridges (Oasis, 6 g, Waters) were conditioned by gravity with 10 mL of methanol and equilibrated twice with 10 mL of filtered seawater. The filtrates were passed through the cartridges at a rate of 1–2 drops per second by reduced pressure. The cartridges were then washed with 10 mL of LC-MS-grade water and eluted with 4 mL of methanol. The samples were dried in a desiccator connected to a vacuum pump, reducing the pressure stepwise to 10 mbar until complete solvent evaporation, and then stored at −80 °C.

### Chromatography and Mass Spectrometry Analysis

For each sample, an LC-MS vial with a septum cap and microinsert was prepared. Each sample was resolubilized in 100 μL of a 1:1 mixture of water and methanol (LC-MS-grade) and vortexed until fully solubilized. Furthermore, a QC pool sample was prepared by pipetting 10 μL of each sample, excluding the blanks, into a single vial. A QC pool blank (seawater samples free of microorganisms) was prepared by combining 1 μL of each blank into a single vial. Ten μL of each mesocosm sample was injected into the UHPLC-HR-MS, and the eluent consisted of high-purity water with 2% acetonitrile and 0.1% formic acid (solvent A) and 90% acetonitrile with 10% water and 1 mmol L^–1^ ammonium acetate (solvent B). The gradient elution was performed using isocratic elution of 100% solvent B for 1 min, followed by a linear gradient from 100% solvent B to 20% solvent B within 5.5 min, a linear gradient from 20% solvent B to 100% solvent B for 0.6 min, and isocratic equilibration at 100% solvent B for 2.9 min. The total runtime was 10 min, and the flow rate was set to 0.6 mL min^–1^.

Mass spectrometry was conducted in positive ionization mode with a scan range of *m*/*z* 75 to 1,125 and a peak resolution of 70,000 for MS1 acquisition. The MS/MS spectra of precursor ions were obtained with the above-mentioned parameters for LC-MS, and within an isolation window of *m*/*z* 0.4, a maximum ion time of 50, and the AGC target was set to 1 × 10^5^.

Metabolome analysis, data processing, and peak deconvolution were done in Compound Discoverer 3.3 (Thermo Fisher Scientific). The analysis workflow consisted of importing raw data for peak picking, deconvolution, and metabolite annotation. Mass tolerance for MS identification was 5 ppm, the minimum MS peak intensity was 2 × 10^5^, and intensity tolerance for isotope search was 30%. The relative standard deviation was set to 50%. The lists of selected labeled compounds were exported as.xlsx files, and their masses were searched against public libraries imported in Compound Discoverer 3.3 (LIPID MAPS, Natural Products Atlas, NIST). The raw LC-MS data were converted into an open-source file format (.mzXML) using the software ProteoWizard.^36^ All files, including mass spectra, Compound discoverer study and results files, and supplementary data Files were uploaded to the public repository MassIVE MSV000097439: MassIVE Data set Summary.

### Data Analysis and Metabolites Identification

The feature data matrix was exported as a.csv file. The intensities were normalized by TIC, log-transformed, and Pareto-scaled in MetaboAnalyst 5.0 ^37^ (https://www.metaboanalyst.ca/MetaboAnalyst/home.xhtml). PCA was performed to compare metabolite similarities between extracts. Assignment of putative identities of significant features detected only in bacteria-treated samples was performed by searching selected MS2 spectra in PubChem using CSI:FingerID^38^ in SIRIUS^38^ and by spectral similarity searches in GNPS.^39^ Analytical standards were purchased, and their fragmentation patterns in MS/MS experiments were compared for full-spectral matching using diagnostic fragments, thereby confirming the annotations at the confidence level of 1.^22^

## Supplementary Material



## Data Availability

The data sets and Supplementary Data File 1 are publicly available at MassIVE spectral database under the name MassIVE **MSV000097439** and the links are https://massive.ucsd.edu/ProteoSAFe/dataset.jsp?task=9b3bef799dc445b084f08bbda0c47e3a; ftp://MSV000097439@massive.ucsd.edu; ftp://massive.ucsd.edu/v09/MSV000097439/.

## Notes

Data that support the findings of this paper, including the samples list with identifier and LCMS data file name for every sample; curated metabolites list; phenotyping and identification of phytoplankton and protozooplankton counted in samples; “List-full”, “List-reduced”, and “List for Script” prepared to run analysis of the plankton composition; “General abundance plot”: the values recorded for Chlorophyll a fluorescence in every samples 24 h after bacteria addition; “Sum of Biovolumes” indicating the normalization factor determined for every sample; the sheet “Comparison_Sum”, “Sum_Phyla_Plot”, and “Chl *a* measurements” containing the Chl *a* values recorded, the biovolumes, and the normalization factor determined for every sample (XLSX)
